# A novel, low-cost microfluidic device with an integrated filter for rapid, ultrasensitive, and high-throughput bioburden detection

**DOI:** 10.1038/s41598-023-38770-x

**Published:** 2023-07-26

**Authors:** Md. Sadique Hasan, Chad Sundberg, Michael Tolosa, Abhay Andar, Xudong Ge, Yordan Kostov, Govind Rao

**Affiliations:** 1 Center for Advanced Sensor Technology, University of Maryland Baltimore County, Baltimore, MD 21250 USA; 2 Department of Computer Science and Electrical Engineering, University of Maryland Baltimore County, Baltimore, MD 21250 USA; 3 Department of Chemical, Biochemical and Environmental Engineering, University of Maryland Baltimore County, Baltimore, MD 21250 USA; 4 Champions Oncology Inc, 855 N Wolfe St, Baltimore, MD 21205 USA

**Keywords:** Lab-on-a-chip, Sensors and probes, Optical spectroscopy

## Abstract

Rapid and accurate bioburden detection has become increasingly necessary for food, health, pharmaceutical and environmental applications. To detect bioburden accurately, and in a highly sensitive manner, we have fabricated a novel microfluidic device with an integrated filter to trap the cells. Bioburden is detected on the filter paper in situ using the redox reaction of fluorescent label resorufin and a portable multichannel fluorometer is used for fluorescence measurement. The microfluidic device was fabricated in a facile, low-cost, and rapid way with microwave-induced thermally assisted bonding. To characterize the bonding quality of the microfluidic cassettes, different tests were performed, and the filter paper material and size were optimized. Primary *Bacillus subtilis* culture bacterial samples were filtered through the device to validate and investigate the performance parameters. Our results show that a limit of detection (LOD) of 0.037 CFU/mL can be achieved through this microfluidic device whereas the LOD in a normal microfluidic cassette in the fluorometer and the golden standard spectrophotometer are 0.378 and 0.128 CFU/mL respectively. The results depict that three to ten times LOD improvement is possible through this microfluidic cassette and more sensitive detection is possible depending on the volume filtered within a rapid 3 min. This novel microfluidic device along with the fluorometer can be used as a rapid portable tool for highly sensitive, accurate and high-throughput bacterial detection for different applications.

## Introduction

Millions of people become ill and die from food, water, or medicine contamination every year^1^. A global disease burden of 45% is attributed to bioburden, according to the World Health Organization^2^. In the manufacturing of biopharmaceuticals, contamination associated with bioburden is also a major concern^3^. As a result, diagnostic devices have become increasingly important for identifying bacteria and their antibiotic susceptibilities^4,5^. For the detection of bioburdens, there are many analytical methods available—adenosine triphosphate bioluminescence^6^, flow cytometry^7^, nucleic acid amplification^8^, respiration^7^, impedance methods^8^, and antibody detection^7^, to mention just a few. Most detection techniques require a relatively long time to detect (3 h to 7 days)^9^. Furthermore, when detection rates are sufficiently fast, cost and sensitivity become limitations^10–12^. Currently, cell viability assays are often used in drug development to study growth factors, cytokines, and cytotoxic agents that are capable of detecting viable cells and bacteria rapidly. In recent years, resazurin has been used in cell viability measurements to measure the oxidation–reduction of a given sample. To detect bioburden rapidly and in a sensitive manner, our previous study^13^ demonstrated the use of a USB-powered portable fluorometer for detecting viable cells in a given sample based on the detection of high quantum yield resorufin from resazurin. In this low-cost system, fluorescence intensity slope served as a criterion for detecting bioburden. Viable cells were monitored using the redox indicator dye resazurin. The sample under investigation was spiked with resazurin and loaded in a special-design microfluidic cassette and the rate of change of resazurin to resorufin was observed via the fluorometer. To detect bioburden with higher sensitivity, a novel microfluidic device with an integrated filter for automated filtration and detection is introduced in this study with the reported fluorometer and assay.

Microfluidics has become a powerful technology in the last few decades and has found applications in several frontier research areas that include analytical chemistry^14^, pharmaceuticals^15^, synthesis of chemicals^16^ and clinical application^17,18^. Microfluidic technologies have also been applied to microorganism studies recently. Microfluidic devices with micro-sized scale and large-scale integration offer many special advantages including low cost, higher throughput, and higher efficiency in microorganism analysis^19–23^ and antibiotic persistence^24^. Recent developments in microfluidics also utilize filter paper as a means of trapping and detecting bacteria and are becoming popular owing to the low-cost, ease of access, processing, modification and disposal^25,26^. However, the reported paper-based microfluidic devices have a very high limit of detection (LOD) ranging from 57 to 500 CFU/mL, and low accuracy^27–30^. The devices often require complex modifications i.e., antibody functionalization, immobilization of nanoparticles and UV-curing for patterns on the filter paper for detection^27–30^. Moreover, the devices are often specific to a certain strain of bacterial detection due to certain antibody functionalization^27–30^. These drawbacks hinder the rapid, facile, low-cost filter paper-based microfluidic devices to be widely used in bacterial detection, especially in field applications.

In this study, we have developed and validated a microfluidic device integrated with filter paper to trap viable bacterial cells initially and detect them finally. An easy, low-cost, rapid and environment-friendly microwave-induced thermally assisted solvent bonding was employed to bond the microfluidic device^31^. Bacterial trapping is done on the filter paper and detection is done in situ. After trapping the cells onto the filter paper, a resazurin-based assay is used as previously reported, and the fluorescence change due to the trapped cells (if any) is monitored using the previously developed multichannel fluorometer^13^. To the best of our knowledge, in this study, we have shown microwave-induced bonding to develop a microfluidic device with integrated filter papers for the first time for bioburden detection.

Different microfluidic layers were designed and fabricated for the accommodation and support of the filter paper inside the device. We have explored different materials and sizes of filter papers and optimized the parameters for higher fluorescence signal attainment and sensitive detection of bioburden. The *Bacillus subtilis* strain was tested to validate the device. *Bacillus* is a widespread, gram-positive bacterium that is harmful to humans, plants, or other organisms and causes severe infectious diseases^32–34^. Different bacterial concentrated samples were prepared and passed through the device at the same amount along with the negative control. Our results show that the integrated filter-based microfluidic cassette demonstrated much higher sensitivity and lower LOD than the gold standard fluorescence measurement device spectrophotometer and a normal microfluidic cassette in the multichannel fluorometer. Also, the LOD can be improved with filtering of higher volume. Sensitivity enhanced by the microfluidic device can lead to higher detection accuracy and detection can be done within a rapid 3 min in the fluorometer after 6 h of incubation of samples*.* Each microfluidic device costs about $1.6 and the 8-channel fluorometer costs approximately $1300 which can be further reduced by scaling up the production and are cost-effective compared to the conventional instruments*.* The facile, easily bonded microfluidic device in combination with the fluorometer can be used for rapid, highly sensitive and accurate detection of bioburden in health, pharmaceutical and environmental applications overcoming the limitations of the previously reported microfluidic devices.

## Methods and materials

### Device fabrication and bonding method

The Polymethyl methacrylate (PMMA) sheets used in this study were laser cut and bonded as described in our previous publication^31^. Briefly, microfluidic channels were cut into substrates according to the requirements. Ethanol was taken in a syringe or micropipette and sprayed on the surface of the sheet to cover the whole surface of the PMMA to be bonded. The PMMA sheets to be bonded were then hand-pressed and then inserted into a Polyetheretherketone (PEEK) vise and the whole setup was placed in the microwave for 1.5 min. The absolute ethanol and microwave heating used for bonding the device are effective for both the sterilization of the filter and the device as well.

**Figure 1:**
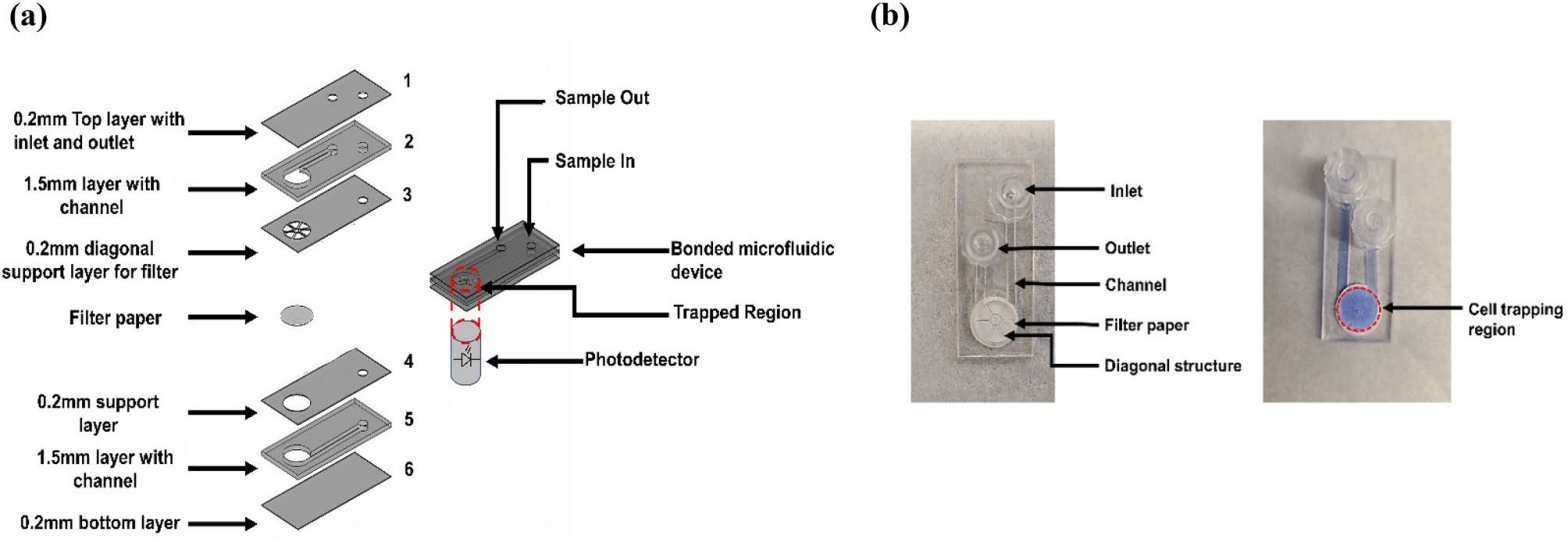
(**a**) Different layers of the microfluidic device integrated with filter paper with corresponding thickness. (**b**) (Left) Photograph of a bonded microfluidic device with different sections, (right) a microfluidic device with the resazurin sample.

The microfluidic device consisted of six different layers of PMMA of different thicknesses of dimensions 50 mm × 22 mm. Figure 1a shows the schematic diagram of different layers used for the fabrication of microfluidic devices with the layer numbers next to it. The top 0.2 mm layer had an opening for the inlet and outlet. Polycarbonate luer locks were used for injecting and taking out the samples. The second layer consisted of a channel to guide the sample out of the device. The third- and fourth layers acted as support for the filter paper with openings or passages for the sample to filtrate and pass through. The third layer also consists of several diagonal structures that support the filter paper and prevent deformation during filtration. The fifth layer had a channel for guiding the sample through the filter paper and the bottom layer formed a seal for the whole device. The filter diameter was 2 mm larger than that of the circular orifice to cover the whole filtration region. Additionally, the filter paper diameter was chosen such that it has an overlap with the inlet and outlet channel to ensure the entirety of the sample is filtered. As the photodetector is located on the bottom side of the device, the cassettes were fabricated and bonded in such a way, that the sample goes through the inlet and passes through the bottom side of the filter first and then goes out of the outlet. In this way, the filter paper does not shade the emission from the fluorescence. For the bonding, each of the layers was wet with ethanol and the filter paper was placed in between the layers as shown in Fig. 1a. Figure 1b shows an actual photograph of the microfluidic cassette with different sections of the cassette labeled. To detect bioburden in the developed microfluidic device, the filter paper does not need any surface modifications making the overall process easier and no deformation was observed in the filter paper after the bonding.

### Filter paper selection

Initially five different filter papers were tested in this study. They are polyethersulfone (PES), polyacrylonitrile (PAN), gray polycarbonate (PC), nucleopore track etched (NTE) and nylon filters. These different filter papers were chosen because of their extensive use for bacterial trapping and detection^35–39^. The different filter papers were tested for autofluorescence in the fluorometer. The filter papers were cut from the filter membrane sheets using a hole punch. Microfluidic devices were bonded using the microwave-induced technique for all the filter papers and deformations and distortions i.e., bending, cracking were noted. For bioburden detection, sterility is an important concern. As the filter papers are not pre-sterilized, they may be susceptible to contamination depending on the storage condition. Therefore, the filter papers were tested for sterility and the best filter paper in terms of sterility and autofluorescence was selected for further experiments.

For sterility testing, 10 mL of negative control Lysogeny broth (LB) was filtered with different non-sterile filters in a manual filter housing. The cells were recovered afterward in 1 mL fresh LB and 200µL of the sample were incubated on the agar plate at 37°c for 24 h and colonies were counted. Additionally, to determine the effectiveness of alcohol sterilization, 5 mL of 70% ethanol was poured into a sterile petri dish. Filters were soaked in 70% ethanol for one minute. Filters were then put in filter holders and washed two times with 10 mL sterile DI water. Afterward, 10 mL of LB was filtered through sterile filters, and cells were recovered in 1 mL fresh LB media and the above procedure was repeated to count the colony forming units.

### Multichannel fluorometer and sample preparation

The details of the multichannel fluorometer are described in the supporting information.

### Microfluidic device tests

The microfluidic devices were tested for leakage and burst upon selecting the filter paper material and diameter. For the burst tests, the inlet of the device was connected to a pressurized airflow that can go up to 60 psi and the outlet was locked. The device was placed under water and the flow was increased by one psi at a time until air bubbles were seen in the water coming from the bonded device and the filter paper was observed for any ruptures and distortions. The same configurations were applied to evaluate the leakage test as well, only the pressure was slightly increased from zero and the cassette was inspected for air bubbles originating from the bonded microfluidic devices. A statistically significant value of the burst pressure and percent of defective devices were obtained by performing the leakage and burst tests ten times each.

Additionally, the pressure buildup profile of the microfluidic device was tested to ensure the filter paper and microfluidic device assembly had the bonding strength to handle the pressure without any deformations during the sample injection and filtration. The pressure drop in the device was evaluated with a negative control LB medium and a highly concentrated (100 CFU/mL) bacterial solution after 10 h of incubation. The pressure was measured using a syringe pump and a pressure sensor assembly. The pressure sensor (PendoTECH PMAT) was placed in between the inlet of the microfluidic device and a syringe pump. The flow of the syringe pump was set at a rate of 5 mL/min and 20 mL of each sample was filtered. The pressure drop was calculated for both samples and compared with the burst test to ensure the proper operation of the filter paper and the device.

### Sample preparation, filtration and fluorescence measurement procedure

The preparation of primary and secondary culture cells is done in the same way as our previous publications^13^. Briefly, a 50 mL primary culture was prepared using 200 µL of *Bacillus* cells, which was grown at 37 °C in a shaker at 150 rpm (Lab-line Instruments, Melrose Park, IL) overnight. The optical density of the primary culture was measured to be 2.5 at 600 nm (SpectraMax M5). The primary seed culture (1%) was used to inoculate into 200 mL secondary culture and was grown at 37 °C in a shaker at 150 rpm to reach an optical density of 0.4 at 600 nm (~ 3 × 108 CFU/mL). This was centrifuged at 5000 RPM (Avanti J-25 I centrifuge, Beckman Coulter, Inc., Brea, CA) for 10 min. The cell pellet was washed with phosphate-buffered saline (PBS, pH 7.2) and the washed cells were re-suspended in PBS, pH 7.2. This sample is used for making serial dilutions (in 50 mL tubes) from 1:10 to 1:105. The 1:105 diluted sample is used to make further dilutions to make a final concentration of viable cells which is calibrated against a standard plate count. For this, the diluted samples from 1000 CFU/mL to 1 CFU/mL were prepared. After preparing different samples, they were incubated in shake flasks at 37 °C for 6 h as optimized in our previous publication^13^. In parallel, 200 µL of each sample was spread on an LB agar plate, and the plates were placed in an incubator at 37 °C overnight. After about 24 h, the number of colonies showing up on the plates was counted. 5 µM resazurin is mainly used in our study for the reduction study. The mechanism of using resazurin for the viability test is given in the supplementary information.

**Figure 2:**
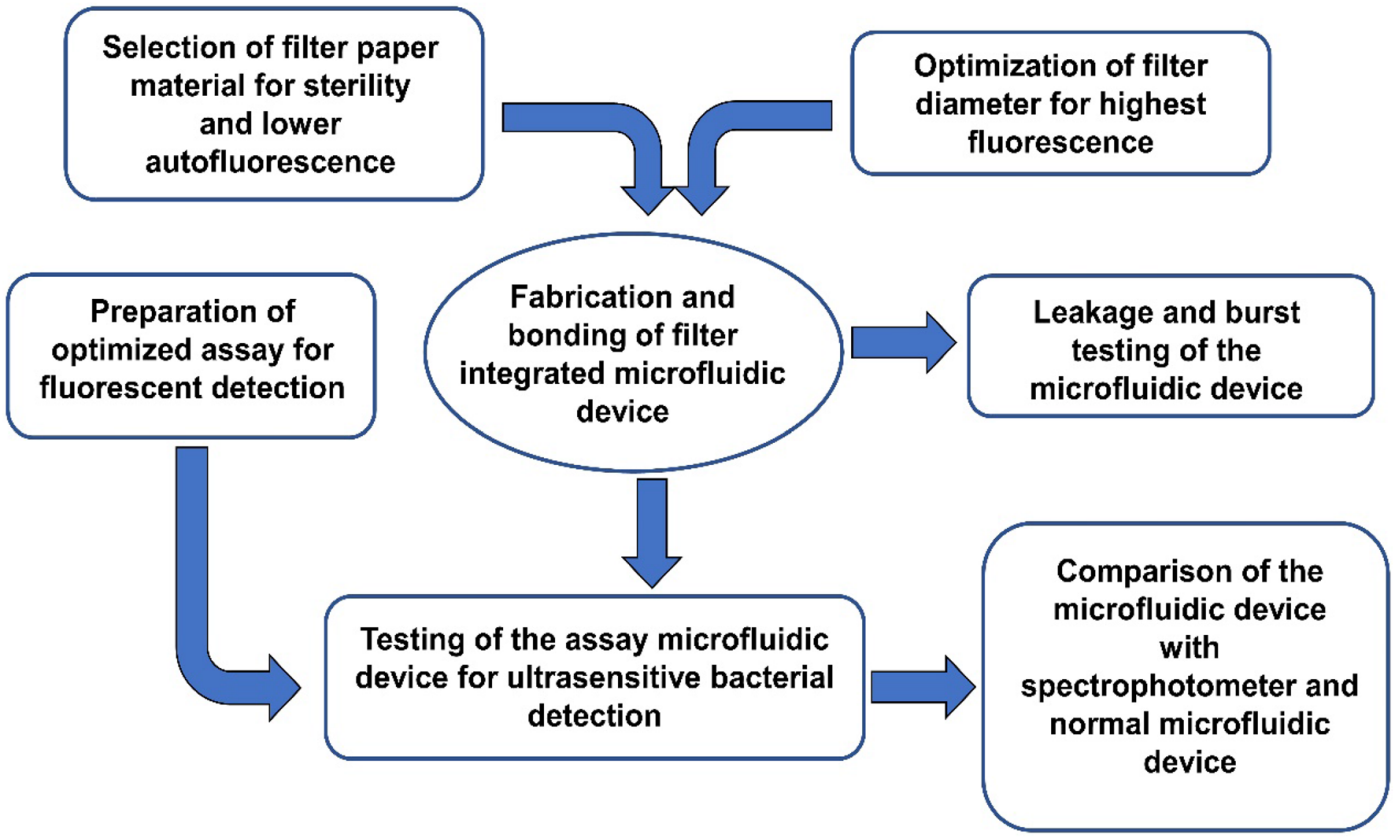
Flow diagram of the performed experimental protocols.

After the incubation, the samples were passed through the inlet of the microfluidic device. A particular volume (initially 1 mL) of the sample was passed slowly through the microfluidic device. Two syringes were used for injecting and backflushing the samples. As there were cavities under and above the filter paper due to the structure, any remaining samples from these regions were flushed out with the syringes. After filtering all the samples, 5 µM resazurin was taken and pushed inside the cassette with a syringe in just the amount (~ 150µL) to be needed to fill the lower side of the filter paper where the cells are trapped. After the resazurin was injected, the cassette was immediately placed in a cassette holder in the multichannel fluorometer, and fluorescence was measured. In parallel, the samples were also tested in the normal microfluidic cassette (Fig. S2) in the fluorometer^6^ and 96-well plates in the spectrophotometer. *Bacillus subtilis* were tested to compare the LOD for the filter-integrated microfluidic device with the normal microfluidic cassettes in the multichannel fluorometer and 96 well plates in the spectrophotometer. Bacterial samples were prepared and tested in two different types of microfluidic cassettes and the spectrophotometer in duplicates*.* Each test was performed with the negative control LB media. The overall experimental protocol is illustrated in Fig. 2.

### Analytical methods

LOD was calculated as the cell concentration that can induce a signal equivalent to 3 times of noise.

1 $${\text{LOD}} = \left( {{3} \times {\text{noise}}} \right)/{\text{sensitivity}}$$

The sensitivity is the average fluorescence slope calculated from the different bacterial samples with negative control LB, assuming LB media containing 0 CFU/mL. The noise is defined as the standard deviation of the intercepts for different response plots of the same experiment performed as the intercept is the fluorometer slope at negative control. The statistical difference between any two samples was calculated with a two-tailed statistical t-test. A p-value of ≤ 0.05 means there is a significant difference between the samples in the test. All the analysis was calculated on 3 min of data for both the spectrophotometer and multichannel fluorometer.

## Results and analysis

### Autofluorescence of the filter paper

As autofluorescence can significantly interfere with the fluorescence measurement, the filter paper with the lowest autofluorescence should be selected for this application. The autofluorescence of different filter papers was evaluated in the multichannel fluorometer with the microfluidic device. Table 1 shows the autofluorescence in the multichannel fluorometer for microfluidic devices with different filter materials. From the table, it can be seen that the gray PC filter has the least autofluorescence and the nylon filter has the highest. The other four materials have quite similar autofluorescence.

**Table 1:** Autofluorescence of the microfluidic device with different filter papers with a filter diameter of 13 mm.

Filter material	Average fluorescence signal (a.u.)	Standard deviation (a.u.)
Nylon	138.1	2.2
PAN	72.7	2.2
Gray PC	56.5	2.1
PES	78.8	2.2
NTE	75.6	2.3

Though having the lowest autofluorescence, the gray PC filter was susceptible to deformation during microwave-induced bonding. Therefore, this filter paper was discarded from the further sterilization test.

### Sterility of different filter papers

The results of the sterility of different filter papers are illustrated in the supplementary information. Based on the results, considering the pre-sterility, autofluorescence and extensive applications, the PES filter was chosen for the microfluidic device.

### Diameter of the filter paper

**Figure 3:**
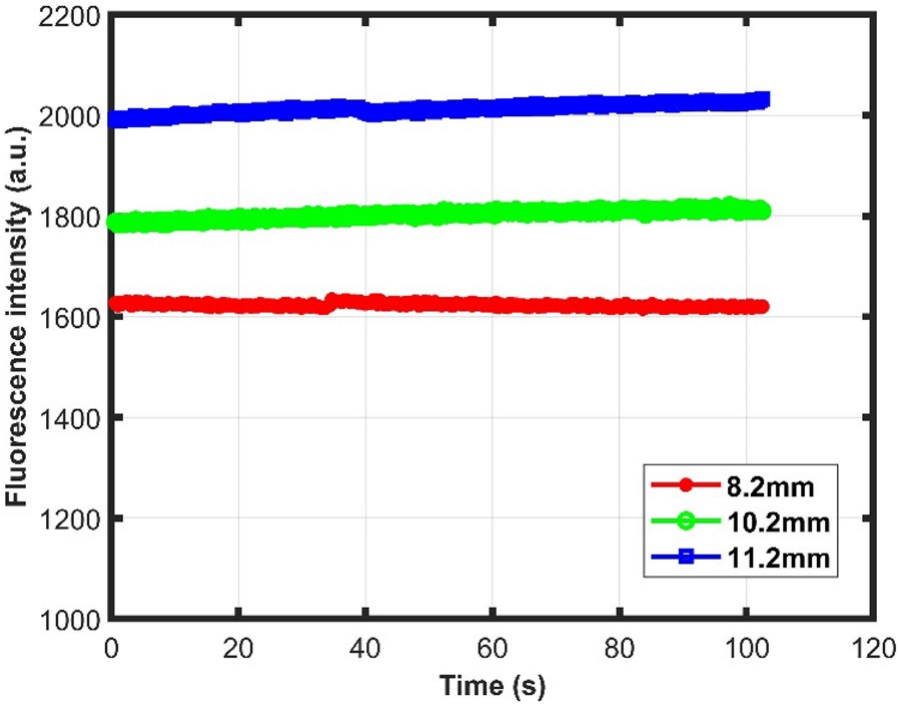
Fluorescence response of PES filter papers with different diameters of the circular orifice.

For the filter paper to cover the entire filtration zone and hold in place, the filter diameter was consistently 2 mm greater than the different diameter circular orifices as mentioned earlier. Three different diameters i.e., 8.2 mm, 10.2 mm and 11.2 mm of the circular opening were tested. The diameter of 8.2 mm was tested as it matches the photodiode opening in the cassette holder. The maximum diameter was set to 11.2 mm as exceeding it would make the filter paper touch the edges of the cassette and result in leaking. The same volume of 0.1 µM resorufin solution was passed through and fluorometer response was recorded. Figure 3 shows fluorescence intensity for varying diameters from the fluorometer. From the figure, the larger diameter opening has higher fluorescence intensity due to the larger area of emission. So, the filter diameter of 13.2 mm was selected for further experiments.

### Leakage and burst test results

The leakage and burst pressure of the whole assembly were tested as previously mentioned. Out of 10 tested microfluidic devices, only 1 out of 10 devices was observed to fail. This is evidence of the reliability of the bonding mechanism and the bonded microfluidic devices. The burst pressure was calculated with and without the filter assembly. Table S2 in the supplementary information shows the average burst pressure of a total of 10 devices.

Additionally, the pressure build-up profile of the whole microfluidic assembly with LB media and a 1000 CFU/mL bacterial sample is shown in supplementary Fig. S3. As can be seen, the maximum pressure drop during filtration of the 20 mL sample is ~ 6 psi which is much less than the average burst pressure (29.2 psi) of the whole assembly and the filter paper. Additionally, for all the burst tests performed, no deformations were seen on the filter paper. So, the microfluidic device bonded using this method is reliable and facilitates the filtration of large volumes without leaking and deformation of the filter paper.

### Results in the fluorometer and LOD comparison

**Figure 4:**
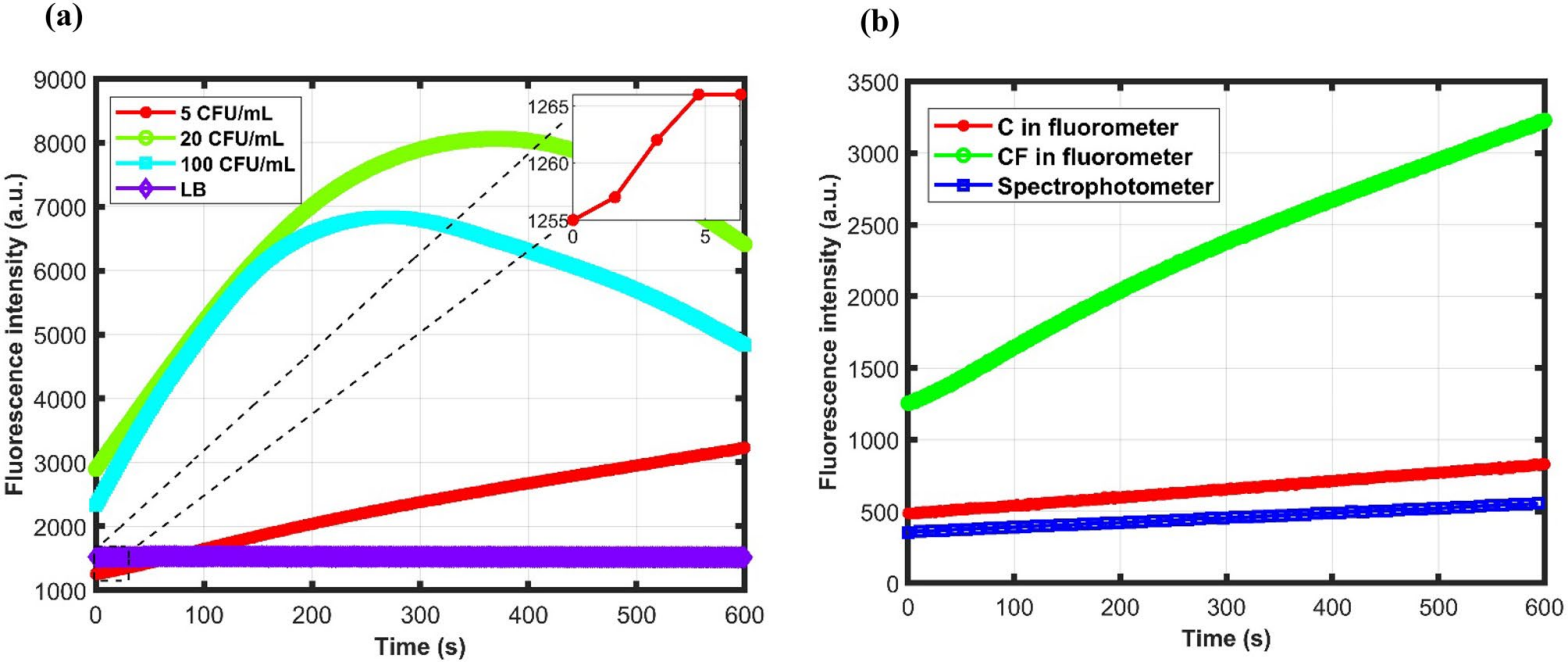
(**a**) Different bacterial sample responses in the fluorometer with the microfluidic cassette with filter (**b**) Response of 5 CFU/mL bacterial sample in two different cassettes in the fluorometer and the spectrophotometer.

Samples were tested in two different microfluidic cassettes in the fluorometer and 96 well in the spectrophotometer to determine the LOD as previously mentioned. Figure 4a shows the response of different bacterial samples with the negative control in the microfluidic device with the integrated filter. The measurements were taken every 1.5 s and the inset shows a part of the 5 CFU/mL sample response. Although all the samples were tested in duplicate, only one sample data is presented graphically. Figure 4b shows the response of 5 CFU/mL bacterial samples in the normal microfluidic cassette(C), microfluidic cassette with filter (CF) in the fluorometer and 24 well plates in the spectrophotometer.

Figure 4a shows that with increasing bacterial load, there is a higher slope observed than that in the LB medium which exhibits very little or no slope. Figure S4 shows the detection region after filtration of LB and bacterial sample and injection of resazurin. For the bacterial sample, the blue dye resazurin is converted to pink resorufin as evident from the figure. For 20 and 100 CFU/mL, a decreasing slope is observed after some time which is due to the reduction of resorufin to colorless dihydroresorufin^40^. The decaying occurs faster and at a lower time for higher concentrations. From Fig. 4b, the fluorescence slope is significantly higher for samples in CF cassettes than from the C cassettes and the spectrophotometer. The slope values are respectively 3.28, 0.57 and 0.33 a.u./s in CF, C in the fluorometer and spectrophotometer which shows a significant increase in the sensitivity with the cassettes integrated with the filter. The average slope values were used to calculate the LOD in different cassettes in the fluorometer and spectrophotometer.

**Figure 5:**
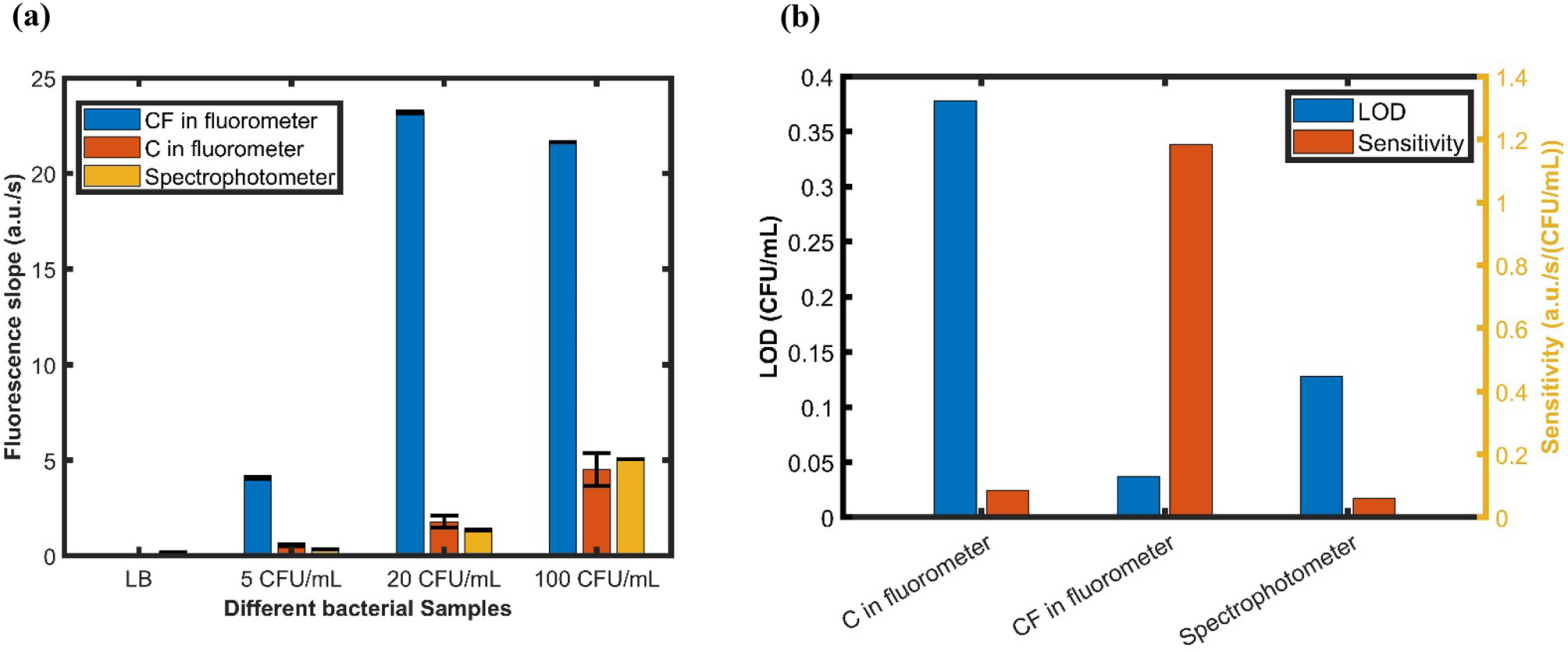
(**a**) Different bacterial sample fluorescence slopes of CF, C in the fluorometer and 96 well plates in the spectrophotometer. (**b**) Calculated LOD and sensitivity of CF, C in the fluorometer and 96 well plates in the spectrophotometer.

Figure 5a shows the fluorescence slope comparison of two different microfluidic cassettes in the fluorometer and the spectrophotometer for different bacterial concentrated samples. Figure 5b Shows the LOD and sensitivity comparison of these samples. The data analysis along with the fluorescence slope for the calculation of sensitivity and LOD are shown in Table 2. The first four rows represent the slope values for different bacterial concentrations along with the negative control.

**Table 2:** Data analysis and sensitivity calculation for the microfluidic cassette with filter (CF), normal cassette (C) in fluorometer and 96 well plates in the spectrophotometer.

CFU/mL	CF in fluorometer		C in fluorometer		Spectrophotometer	
	1	2	1	2	1	2
0	0.045	0.045	0.035	0.012	0.210	0.210
5	4.088	3.984	0.562	0.467	0.339	0.335
20	23.203	23.098	1.786	2.411	1.345	1.373
100	21.640	21.644	4.522	6.256	5.049	5.049
Sensitivity (0–20)	1.185	1.181	0.086	0.122	0.059	0.061
Intercept	0.761	0.790	0.076	0.055	0.138	0.133
Average Sensitivity (0–20)	1.183		0.086		0.060	
Noise	0.014		0.011		0.003	
LOD	0.037		0.378		0.128	
t-test (LB vs 5)	0.008		0.050		0.010	
t-test (LB vs 20)	0.001		0.023		0.008	
t-test (LB vs 100)	0.000		0.025		0.001	

Data analysis shows a significant difference between the LB and bacterial samples.

From Fig. 5a, the slope values of LB media for all the samples are significantly smaller than the other bacterial concentrated samples. This is also shown by the p-values of the statistical t-test in Table 2. Also, for increasing bacterial concentrations the slope values are higher for all the samples except for the 100 CFU/mL sample in CF which is due to dihydroresorufin conversion. Nevertheless, the slope values for samples in CF are significantly higher than the other two samples for all cases. So, the CF microfluidic cassettes are significantly more sensitive than the normal microfluidic cassettes and the spectrophotometer. Figure 5b illustrates the results of the sensitivity and LOD for all the samples calculated for the linear region. From the results, the cassettes with integrated filter (CF) in the fluorometer are much more sensitive than the other two types of samples resulting in much lower LOD. The LOD is improved ~ 3.5 times than the spectrophotometer and 10 times than the C in the fluorometer. The p-value of the t-test between the sensitivity values of CF and C is 0.0097 and CF and spectrophotometer is 0.000079 which shows that the sensitivity is statistically significantly higher for the CF. Consequently, the samples in CF are around 14 and 19 times more sensitive than C in the fluorometer and spectrophotometer respectively. The exact values are depicted in Table 2. The sensitivity was calculated using the linear range (first 3 min) of responses using 0 to 20 CFU/mL data as the 100 CFU/mL exhibited non-linear response.

### Change of sensitivity with higher filtrate volume

**Figure 6:**
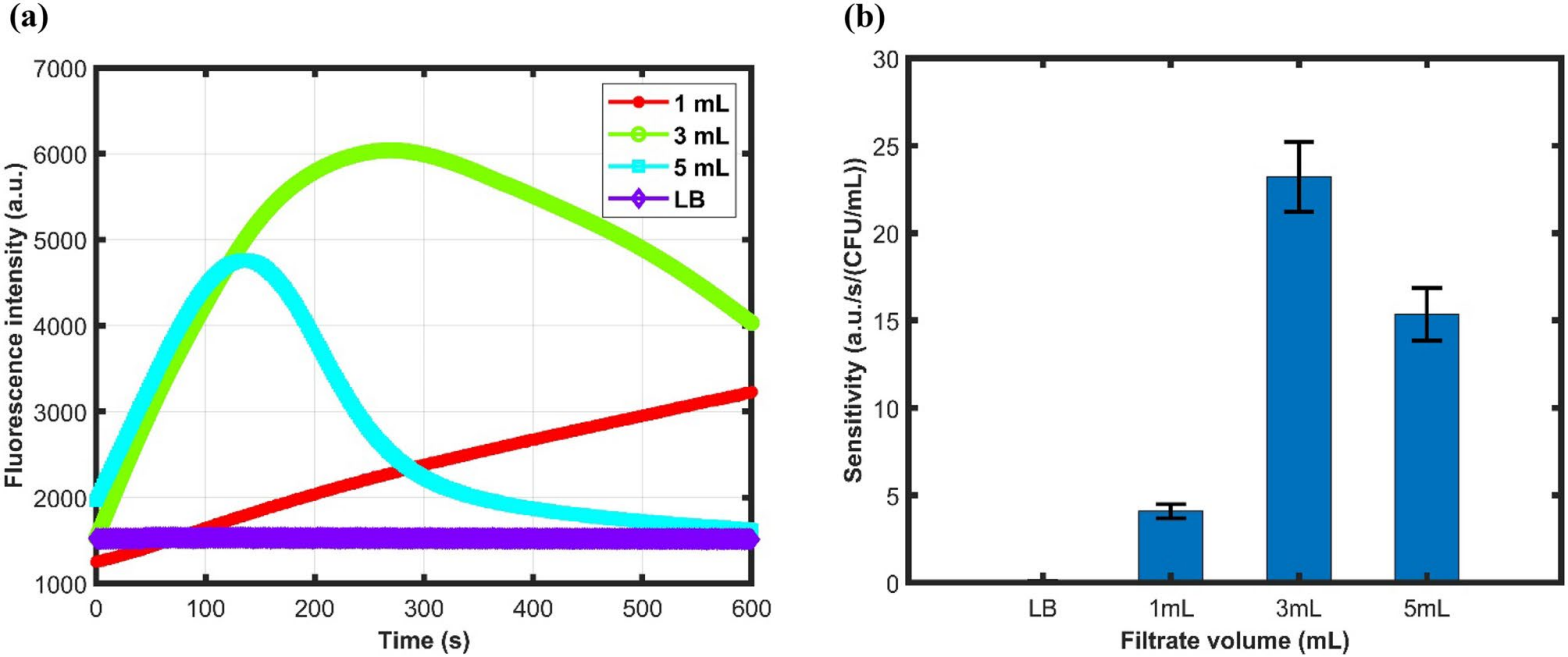
(**a**) Fluorometer response with the microfluidic cassettes with filter for varying filtered volume of 5 CFU/mL *Bacillus* (**b**) Sensitivity of the microfluidic device with increasing filtrate volume along with the negative control LB.

Different volume of the same concentrated bacterial sample (5 CFU/mL) was passed through the cassette to compare the LOD with varying volumes of 1, 3 and 5 mL. The sample response for different filtered volumes is shown in Fig. 6a. The sensitivity comparison for varying volumes is shown in Fig. 6b.

From the figure, it is observed that with increasing volume filtered through the microfluidic device, the fluorescence intensity and slope increase significantly. The increase in turn enhances the sensitivity of the microfluidic device with the fluorometer as shown in Fig. 6b. With more volume filtered, more cells were trapped on the filter paper. This caused resazurin to be reduced to resorufin more rapidly. For higher volumes filtered, in this case, for 5 mL, the sensitivity or average fluorescence slope is decreased. This occurs as a result of decreasing slope after some time which is due to the reduction of resorufin to colorless dihydroresorufin which is evident from Fig. 6a. A similar change of response and sensitivity augmentation with increasing volume for 20 CFU/mL is shown in Figure S5. Nevertheless, the fluorescence intensity and sensitivity are significantly higher than negative control LB media which confirms the presence of bioburden. It is possible to filter more volumes by this microfluidic device to detect very low bioburden concentrations when applicable.

Our device and method can detect bacteria within 6 h after incubation with laboratory cultured samples, reducing detection time by at least four times than the golden standard culture method. The comparatively rapid methods like PCR, ELISA, and other biosensing methods have the disadvantage of low sensitivity, false positive detection, technical complicated procedure and high cost. Following incubation, sample injection takes only one minute depending on volume and can be done easily. Whereas other bacterial detection procedures i.e., PCR, ELISA, and bacterial culture methods require certain procedures, technical preparation and extremely clean operating areas which are more complicated and require trained personnel to complete. This is where our microfluidic device with the fluorometer outperforms other conventional methods for detecting ultra low-level bioburdens. The optimized assay can be used with this device to detect other strains with higher sensitivity as well.

## Conclusion

There is an inherent need for rapid and sensitive bioburden detection in a variety of applications, including public health. To detect bioburden with higher sensitivity and accuracy, a novel microfluidic device has been fabricated and bonded in a facile, environmentally friendly way in this study. Microfluidic cassette parameters have been optimized in conjunction with the multichannel fluorometer for ultra-sensitive detection. The microfluidic device has proven effective for detecting bioburden with a much higher sensitivity than a normal microfluidic device reported before and the spectrophotometer. Our future studies will include testing the microfluidic device with other bacterial strains, real-world assays, filtration efficiency of filter paper and implementing different biodegradable materials for fabrication and bonding. The microfluidic device can have a far-reaching impact on detecting bioburden, especially in a point-of-care setting.

### Supplementary Information

Supplementary Information.

## Data Availability

All data generated or analyzed during this study are included in this published article and its supplementary information files.
