# Author Correction: A novel, low-cost microfluidic device with an integrated filter for rapid, ultrasensitive, and high-throughput bioburden detection

**DOI:** 10.1038/s41598-023-40387-z

**Published:** 2023-08-15

**Authors:** Md. Sadique Hasan, Chad Sundberg, Michael Tolosa, Abhay Andar, Xudong Ge, Yordan Kostov, Govind Rao

**Affiliations:** 1 Center for Advanced Sensor Technology, University of Maryland Baltimore County, Baltimore, MD 21250 USA; 2 Department of Computer Science and Electrical Engineering, University of Maryland Baltimore County, Baltimore, MD 21250 USA; 3 Department of Chemical, Biochemical and Environmental Engineering, University of Maryland Baltimore County, Baltimore, MD 21250 USA; 4 Champions Oncology Inc, 855 N Wolfe St, Baltimore, MD 21205 USA

Correction to: *Scientific Reports* 10.1038/s41598-023-38770-x, published online 26 July 2023

The original version of this Article contained an error in the spelling of the author Abhay Andar which was incorrectly given as Abhay Andara.

In addition, the zip code in affiliations 1, 2 and 3 was incorrect. The correct affiliations are listed below.

1. Center for Advanced Sensor Technology, University of Maryland Baltimore County, Baltimore, MD 21250, USA.
2. Department of Computer Science and Electrical Engineering, University of Maryland Baltimore County, Baltimore, MD 21250, USA.
3. Department of Chemical, Biochemical and Environmental Engineering, University of Maryland Baltimore County, Baltimore, MD 21250, USA.

The original Article and accompanying Supplementary Information file have been corrected.

