# Supplementary material for: A novel, low-cost microfluidic device with an integrated filter for rapid, ultrasensitive, and high-throughput bioburden detection
Source: Sci Rep. 2023 Jul 26;13:12084. doi: 10.1038/s41598-023-38770-x (PMC10372024; doi:10.1038/s41598-023-38770-x)
Supplement: Supplementary file 1 — Supplementary Information. [file 41598_2023_38770_MOESM1_ESM.docx]

**Supporting Information: A novel, low-cost microfluidic device with an integrated filter for rapid, ultrasensitive, and high-throughput bioburden detection**

Md Sadique Hasan^1,2^, Chad Sundberg^1,3^, Michael Tolosa^1^, Abhay Andar^4^, Xudong Ge^1,3^, Yordan Kostov^1^, Govind Rao^1,3*^

*^1^ Center for Advanced Sensor Technology, University of Maryland Baltimore County, Baltimore, Maryland, 21250, USA*

*^2^ Department of Computer Science and Electrical Engineering, University of Maryland Baltimore County, Baltimore, Maryland, 21250, USA*

*^3^ Department of Chemical, Biochemical and Environmental Engineering, University of Maryland Baltimore County, Baltimore, Maryland, 21250, USA*

^4d c^ Department of Chemical, Biochemical and Environmental Engineering, University of Maryland Baltimore County, Baltimore, Maryland, 2127, USA

*Champions Oncology Inc, 855 N Wolfe St, Baltimore, Maryland, 21205, USA*

** Email:* [*grao@umbc.edu*](mailto:grao@umbc.edu)

**Table of Contents**

**Supplementary Section. Methods and Materials**

Subsection. Multichannel fluorometer

Subsection. Detection machanism

**Supplementary Section. Results and analysis**

Subsection. Sterility of different filter papers

**Figure S1.** The multichannel fluorometer in operation for bioburden detection.

**Figure S2.** A photograph of the PMMA microfluidic cassette.

**Figure S3.** Pressure buildup profile during the injection and filtration of LB and 1000 CFU/mL bacterial media with a pressure sensor and syringe pump.

**Figure S4.** Image of the detection region after filtration of LB and bacterial media and injection of resazurin.

**Table S1.** The bacterial colonies formed after 24 hours of incubation for each filter paper material before and after sterilization.

**Table S2.** Average burst pressure of a total of 10 microfluidic devices with the percentage of defective devices.

**Supplementary Section. References**

**Methods and materials**

**Multichannel fluorometer**

The multichannel fluorometer’s circuitry is designed inside the same manner as the sooner in-residence designed single-channel fluorometer [1,2] with adjustments to read a better signal-to-noise ratio and boost the sensitivity and as mentioned in the previous publication[3]. In short, the multichannel device can be used to perform duplicates (or triplicates) for the confirmation of the exams. The device capabilities a semiconductor light source for fluorescence excitation (Green LED, middle wavelength 525 nm, FWHM 20 nm) and its emission is filtered with a bandpass interference clear out with a center wavelength of 532 nm and bandwidth of forty nm. The LED is driven by means of a voltage-controlled current source that could output as much as 50 mA DC. The fluorescence is detected by a photodiode mounted at 90° to the excitation beam, with the emission filter (600 ± 20 nm) installed in the front of it. The channels communicate with a single laptop computer through USB, and they're powered from the computer. The computer software is written in LabVIEW. The initiation of measurement and recording of the fluorescence modulation can be controlled via this system. The computer software addresses the boards via the hub board and a set of multiplexers. The cassette holder is cut out from layers of black PMMA and fixed collectively. The cassette holder is designed to help narrower cassettes that can be illuminated from the slender aspect and deliberately made taller to keep away from the direct illumination of the emission filter out at a shallow perspective via reflections. **Figure S1** shows a multichannel fluorometer in operation.

**Detection Mechanism**

In every living cell, NADH serves as "energy currency". Hence, the presence of continuous NADH production suggests that live metabolizing cells are present. Resazurin is an indicator dye that is directly reduced by NADH. Resazurin is readily absorbed through the cell wall by the cells and exhibits low toxicity. Resorufin, the product of this reaction, is spectroscopically different and highly fluorescent (quantum yield = 0.97) The concentration of the resorufin gradually increases with time, thus acting as an amplification mechanism that is used for bioburden detection.

**Results and discussion**

**Sterility of different filter papers**

As the filter papers do not come pre-sterilized, it is necessary to sterilize them from bioburden. The sterility of different filter paper was tested using negative control LB media with and without sterilization using 70% ethanol as mentioned previously. The bacterial colonies were calculated after 24 hours of incubation for each filter paper material and the results are listed in supplementary Table **S1**. From the results, non-sterile and ethanol-sterilized PES filters are the most sterile.

**
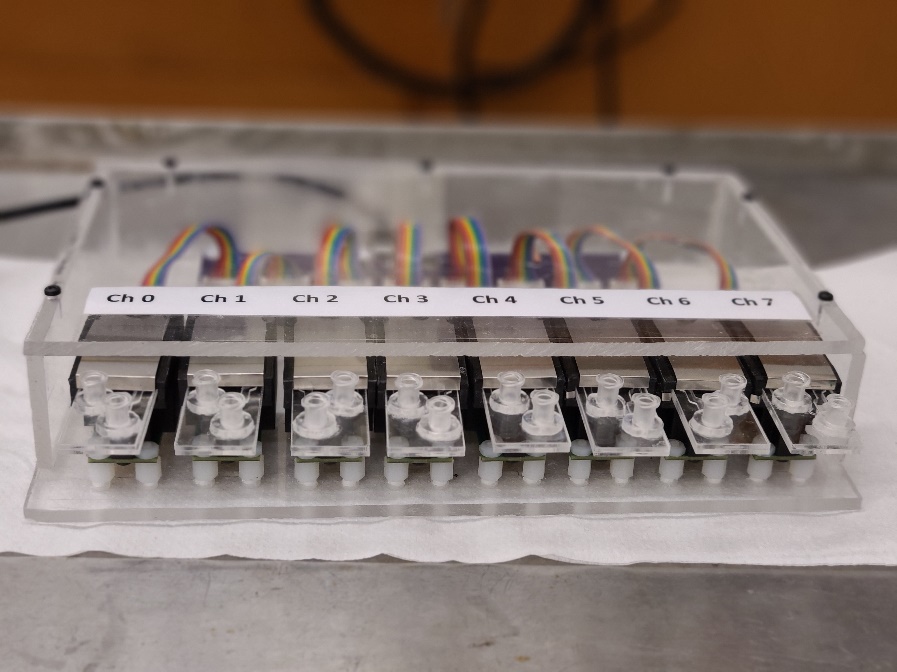
**

**Figure S1.** The multichannel fluorometer in operation for bioburden detection


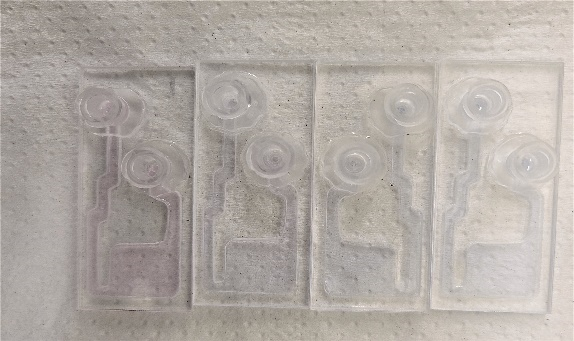


**Figure S2.** A photograph of the normal PMMA microfluidic cassettes.

**
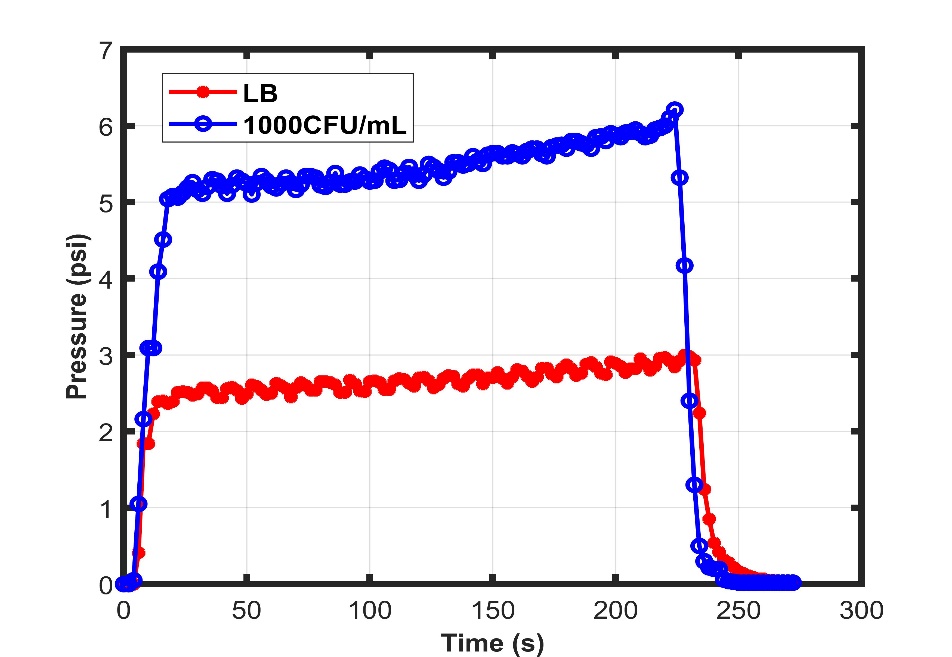
**

**Figure S3.** Pressure buildup profile during the injection and filtration of LB and 1000 CFU/mL bacterial media of 20 mL after 10 hours of incubation with a pressure sensor and syringe pump.

**
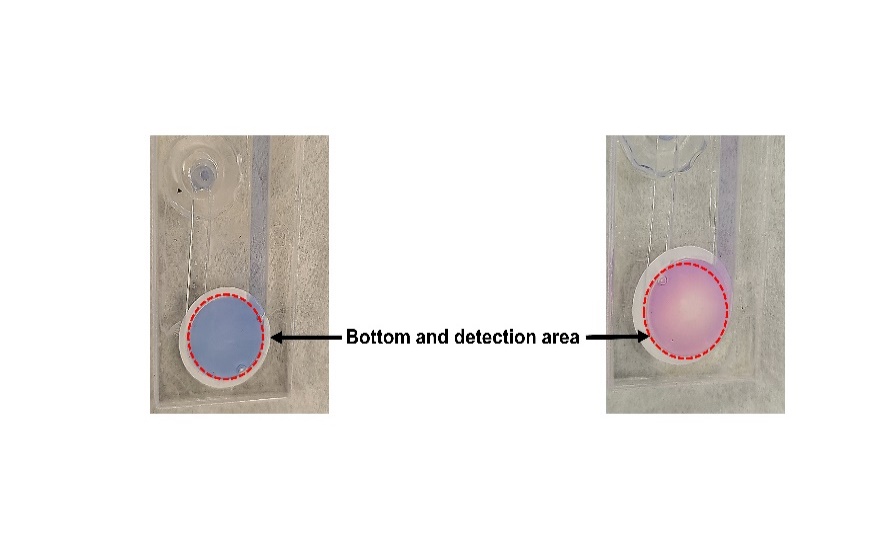
**

**Figure S4.** Image of the detection region after filtration of LB and bacterial media and injection of resazurin. For bacterial media, the blue dye resazurin is converted to pink resorufin.


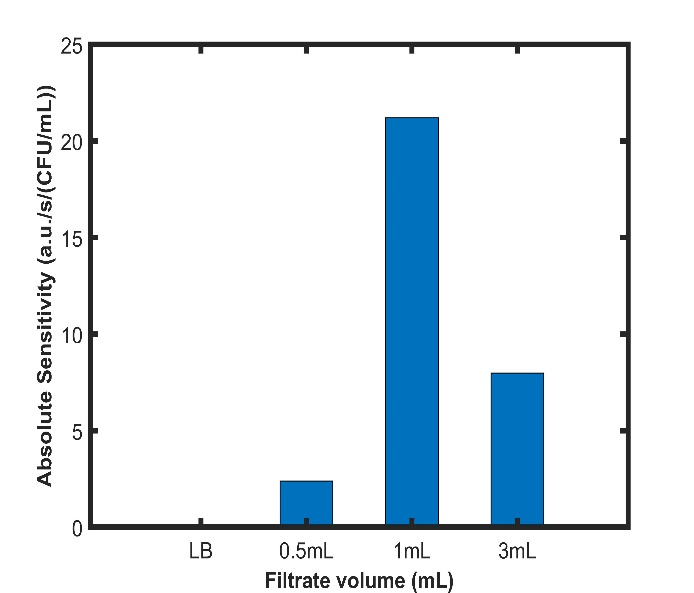

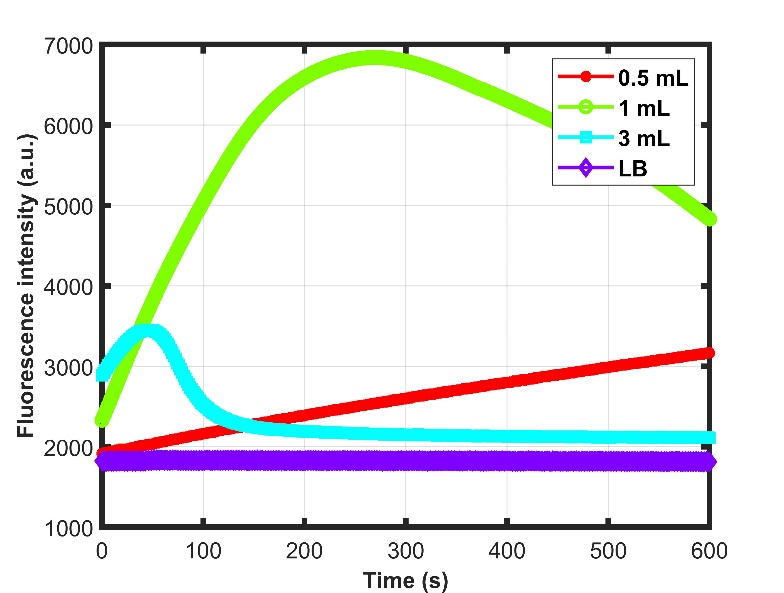


**Figure S5.** a) Fluorometer response with the microfluidic cassettes with filter for varying filtered volume of 20 CFU/mL *Bacillus* strain b) Sensitivity of the microfluidic device with increasing filtrate volume along with the negative control LB.

**Table S1.** The bacterial colonies formed after 24 hours of incubation for each filter paper material before and after sterilization for a filter diameter of 13 mm. The PES filter material is the most sterile.

| **Filter material** | **Blank** | **Ethanol Sterilized** |
| --- | --- | --- |
| PES | 0 | 0 |
| PAN | 10 | 0 |
| NTE | 5 | 0 |
| Nylon | 12 | 3 |

**Table S2**. Average burst pressure of a total of 10 microfluidic devices with the percentage of defective devices.

|  | **Average burst pressure (psi)** | **Standard deviation (psi)** | **Percent defective (%)** |
| --- | --- | --- | --- |
| **10 Total Devices** | 29.2 | 5.83 | 10 |

**References:**

(1) Gurramkonda, C.; Mupparapu, K.; Abouzeid, R.; Kostov, Y.; Rao, G. Fluorescence-Based Method and a Device for Rapid Detection of Microbial Contamination. *PDA Jour.of Pharma. Science and Tech.* *68* (2), 164–171 (2014).

(2) Al-Adhami, M., Tilahun, D., Rao, G., Gurramkonda, C., & Kostov, Y. Rapid detection of microbial contamination using a microfluidic device. *Biosensors and Biodetection: Methods and Protocols Volume 1: Optical-Based Detectors*. 287-299 (2017).

(3) Hasan, M. S., Marsafari, M., Tolosa, M., Andar, A., Ramamurthy, S. S., Ge, X., ... & Rao, G. Rapid Ultrasensitive and High-Throughput Bioburden Detection: Microfluidics and Instrumentation. *Analytical Chemistry*. 94(24), 8683-8692 (2022).
